# Effectiveness of a healthy lifestyle promotion program as adjunctive teletherapy for treatment-resistant major depression during COVID 19 pandemic: A randomized clinical trial protocol

**DOI:** 10.1097/MD.0000000000022958

**Published:** 2020-11-06

**Authors:** Capilla Navarro, Aina M. Yáñez, Aurora Garcia, Andrea Seguí, Francisco Gazquez, Jose Antonio Marino, Olga Ibarra, Maria J. Serrano-Ripoll, Rocio Gomez-Juanes, Miquel Bennasar-Veny, Joan Salva, Bárbara Oliván, Miquel Roca, Margalida Gili, Mauro Garcia-Toro

**Affiliations:** a Research Institute of Health Sciences (IUNICS-IDISBA); b Department of Nursing and Physiotherapy and Research Group on Global Health and Human Development; c Primary Care Research Unit of Majorca, Balearic Islands Health Services and Department of Psychology, University of the Balearic Islands, Palma; d Department of Psychology and Sociology, University of Zaragoza and Preventive Activities and Health Promotion Network, REDIAPP (G06/170), Zaragoza, Spain.

**Keywords:** COVID-19, mindfulness-based cognitive therapy, multicomponent lifestyle program, treatment-resistant depression

## Abstract

**Introduction:**

Treatment-resistant depression (TRD) has a high prevalence and can be exacerbated by poor physical health and economic hardships, which have become common stressors during the current COVID-19 pandemic. The therapeutic approaches used to treat these patients are not always available, may be not be accepted by some patients, and often require face-to-face interactions.

**Objective:**

The main aim of this study will be to evaluate the effectiveness of an Internet-based adjuvant lifestyle-based intervention for patients with TRD.

**Methods:**

This will be a parallel, randomized, and controlled clinical trial. A total of 180 patients with TRD will be randomly allocated (1:1:1) to 1 of 3 groups: treatment prescribed by the mental health team and written suggestions for lifestyle changes (placebo control group); treatment prescribed by the mental health team, written suggestions for lifestyle changes, and an 8-week mindfulness-based cognitive therapy program (active control group); or treatment prescribed by the mental health team, written suggestions for lifestyle changes, and an 8-week lifestyle change promotion program (intervention group). We will perform this study during the COVID-19 pandemic, and will administer interventions by teletherapy, and contact participants by telephone calls, text messages, and/or teleconferences. We will collect patient data using questionnaires administered at baseline, immediately after the intervention, and after 6 and 12 months. The primary outcome will be score on the Beck Depression Inventory-II. The secondary outcomes will be score on the Clinical Global Impressions Scale (used to quantify and track patient progress and treatment response over time) and health-related quality of life measured using the European Quality of Life-5 Dimensions Questionnaire.

**Discussion:**

Patients with TRD are especially vulnerable when face-to-face psychotherapy is unavailable. The main strength of the proposed study is the novelty of the intervention to be used as an adjuvant therapy. Our results may provide guidance for treatment of patients with TRD in future situations that require lockdown measures.

**ClinicalTrials registration number:**

NCT04428099

## 1 Introduction

Major depression is a highly prevalent condition and the second leading cause of disease-induced disability in Spain,^[1,2]^ even though there are effective biological, psychosocial, and other therapeutic treatments. Unfortunately, treatments are not always available, may not be accepted by patients, and may not provide satisfactory results.^[3,4]^ Treatment of patients with depression has become more difficult during the COVID-19 pandemic. Treatment-resistant depression (TRD) is a controversial construct,^[5,6]^ but a commonly accepted definition is: “an incomplete or inadequate response to at least 2 pharmacological treatments, where patients continue to meet DSM-5 criteria for major depression 1 month after treatment.”^[7]^

It is therefore necessary to identify therapeutic alternatives for patients with TRD, such as combinations of standard treatments, without diminishing tolerance and safety.^[8]^ There is some evidence that interventions based on improving lifestyle may be effective for patients with TRD.^[9]^ Lifestyles are changing quickly in developed countries, and the appearance and persistence of depression are related to a variety of biological and psychosocial factors that are related to lifestyle. These include physical inactivity, unhealthy diet, insomnia, social withdrawal, and excessive stress.^[4,8,10]^ Therefore, an intervention that promotes lifestyle improvements may be helpful for patients with TRD.^[11–16]^

In addition to investigating the effect of modification of individual lifestyle factors, there has been progress in studying modification of combinations of lifestyle factors as a therapeutic tool for various medical conditions and mental disorders, such as depression.^[17–20]^ We recently tested a multimodal program that includes interventions that are particularly effective for patients with depression: daily physical activity, adherence to a Mediterranean diet, sleep hygiene, and regulated exposure to sunlight.^[21]^ Other studies also examined the effect of other interventions, such as promoting sociability and teaching emotional self-control techniques, that should also be considered.^[22,23]^ Some researchers proposed this approach as an alternative to psychotherapy or pharmacotherapy as the first treatment for depression.^[9]^ There is evidence that the clinical manifestations of depression decreased at 2 to 6 months after patients followed specific recommendations regarding improvements to lifestyle.^[9]^ However, this benefit subsequently disappeared, probably because of the limited duration of support, monitoring, and supervision.^[22]^ Group therapy has the potential to promote long-term adherence to an intervention program and improve patient outcome.^[24]^

Extensive scientific evidence indicates that online treatments for affective disorders are effective.^[25]^ The benefits of these treatments, compared with usual treatments, show a clear need to consider this approach in treatment guidelines for depression, regardless of symptom severity.^[26]^ Online treatments and teletherapy could therefore be a cost-effective alternative to traditional psychological care for the prevention of relapses and depressive episodes.^[27]^ The “Smile is Fun” program is an example of an effective online intervention that helped patients with moderate symptoms of stress and depression.^[27]^

Previous research of online psychological treatments mainly focused on Internet-based cognitive behavioral therapy (iCBT), and found that this was an effective and practical alternative to face-to-face interventions for treatment of depression.^[28]^ Some studies also demonstrated the effect of practicing mindfulness as an online treatment for depression.^[27]^ Moreover, a recent study reported that the combination of practicing mindfulness and iCBT (iMBCT) had potential for treatment of depression.^[29]^

One of the main challenges of Internet-based clinical interventions is the difficulty in establishing an adequate therapeutic bond, an essential component for successful psychological interventions. However, previous studies suggested that online therapies can establish satisfactory therapeutic alliances.^[30]^ Despite the great advances in the online treatment of depression, many aspects of these interventions require further study, such as the efficacy of online multimodal lifestyle programs for patients with TRD.

There is little scientific evidence supporting the benefits of an online adjuvant healthy lifestyle promotion program for patients with TRD, probably because implementation of these interventions is difficult.^[31]^ There is evidence that psychotherapy delivered by telephone or online may be useful for patients with depression, but less information is available regarding the use of Internet-based programs that focus on lifestyle in health emergency situations in which people are required to restrict their mobility and maintain physical distance.^[32,33]^ As far as we are aware, no previous studies have examined the effect of this approach for patients with TRD.

The main objective of this study is to analyze the effectiveness of treatment prescribed by the mental health team, written suggestions for lifestyle changes, and an 8-week lifestyle change promotion program in patients with TRD. There will be 2 control groups: a placebo control group will receive treatment prescribed by the mental health team and written suggestions for lifestyle changes; an active control group will receive treatment prescribed by the mental health team, written suggestions for lifestyle changes, and an 8-week iMBCT program, an intervention that has proven effectiveness in a face-to-face format.^[34,35]^

We hypothesize that patients randomized to the lifestyle intervention group will show significantly greater improvements in clinical measures of depression than patients from the 2 control groups at 12 months.

## 2 Methods

### 2.1 Study registration

The protocol for this study was registered with the ClinicalTrials.gov registration number: NCT04428099 on June 11, 2020 (version 1.0). Available online: https://clinicaltrials.gov/ct2/show/NCT04428099

### 2.2 Ethics committee

Ethics approval was granted by the Research Ethics Committee of the Balearic Islands (project approval number IB3925/19PI; approved May 29, 2019). The study has been developed in accordance with the Helsinki Declaration. All subjects will sign an informed consent form before group allocation. Their data will be anonymized and will only be used for the purposes of the study. Both participants and healthcare professionals will be informed of the results. Patients allocated to the control group will have the opportunity to follow the intervention group recommendations once the study is finished, if the final results recommend doing so. The Research Ethics Committee will be notified of any protocol modifications.

### 2.3 Research design

This parallel, randomized, controlled trial will be a pragmatic clinical trial conducted under “real world” conditions. We will recruit patients from the Balearic Islands (Spain) who have experienced an episode of TRD. This research protocol has been prepared according to the SPIRIT statement.^[36]^

### 2.4 Participants

#### 2.4.1 Inclusion criteria

The inclusion criteria are age 18 years or older; male or female; diagnosis of major depressive disorder based on DSM-5 and Mini-International Neuropsychiatric Interview (MINI) criteria and at least 2 failed attempts at psychopharmacological treatment for the episode; ability to understand written and spoken Spanish; sufficient physical and cognitive aptitude to understand and provide written informed consent; and access to technologies and knowledge needed to engage in online videoconferences at home, with assistance from family members if necessary.

#### 2.4.2 Exclusion criteria

The exclusion criteria were age less than 18 years; non-Spanish speaking; presence of another disease that affects the central nervous system, such as organic brain pathology, traumatic brain injury of any severity, or dementia; another psychiatric diagnosis or serious psychiatric illness, such as (substance dependence or abuse, history of schizophrenia or other psychotic disorders, eating disorders (except for anxiety or personality disorders based on medical history and MINI criteria):^[37]^ presence of a serious or uncontrolled medical, infectious, or degenerative illness that may interfere with affective symptoms; presence of delirium or hallucinations; risk of suicide; pregnancy or breast-feeding; and the presence of any medical, psychological, or social problem that could seriously interfere with participation in the study.

### 2.5 Intervention

Patients will be randomly allocated to 1 of 3 groups:

1. Intervention group: Treatment prescribed by the mental health team, written suggestions for lifestyle changes, and an 8-week lifestyle change promotion program.
2. Placebo-control group: Treatment prescribed by the mental health team and written suggestions for lifestyle changes.
3. Active-control group: Treatment prescribed by the mental health team, written suggestions for lifestyle changes, and an 8-week iMBCT program.

The eight 3-hour group sessions administered to the intervention group (Table 1) will include review and assignment of homework on the following topics about depression and healthy lifestyles: symptoms, causes, course, and treatment of depression; importance of physical exercise for physical and mental health; how to exercise safely and comfortably; motivation to incorporate physical exercise into daily routines; importance of good nutrition for improving physical and mental health, and healthy eating guidelines; importance of a social support network, with practical proposals for improvements; sleep-wake rhythm and sleep hygiene, with practical proposals for improvement; need for appropriate exposure to sunlight, with practical recommendations; role of negative ruminations in maintaining depression, with strategies to detect and stop this behavior; contact with nature, with practical recommendations for improvements. All strategies will be reviewed and there will be a final opportunity to recommend their combined long-term use.

**Table 1:** Sessions descriptions for the intervention group and the active control group.

	Lifestyle change sessions (intervention group)		Mindfulness-based cognitive therapy sessions (active control group)	
		
Session	Topic	Homework	Topic	Homework
1	Symptoms, causes, course, and treatment of depression. Importance of physical exercise for physical and mental health. Education on how to exercise safely and comfortably. Motivation to incorporate physical exercise into daily routines.	If physically able, complete an aerobic exercise plan (at least 30 min, 3 times per week), which will be increased over time in a personalized manner.	Symptoms, causes, course, and treatment of depression. Concept of full attention. Going from living on “automatic pilot” to living consciously and deliberately. Observe breathing and the body. Raisin exercise.	Attention to breathing, 24 min with audio every day. Record the facets of life in which you are not present (brief). Choose a routine task to perform with full attention, such as the raisin exercise. Mindful eating.
2	Importance of good nutrition in improving physical and mental health. Education on healthy eating guidelines, with a focus on the Mediterranean diet.	Keep a daily food record to determine the need for dietary changes.	Change the form of relating to experiences through thought to feeling them directly. Label the experiences (pleasant, unpleasant).	Body scan, 40 min with audio every day. Focusing on breathing, 10 min with audio several times. Complete the record of pleasant experiences (one per day, specifying the thoughts, emotions, and sensations associated with the experience). Choose a routine task to perform mindfully this week.
3	Importance of relationships. Education on how to improve the social support network, with practical proposals.	Schedule at least 3 face-to-face or online meetings with people who are emotionally close and then increase the number of these meetings over time, if possible.	Change from delving into the past and anticipating the future to being fully present in the moment.	Conscious stretching (even days). Body scan (odd days). Record unpleasant experiences (analogous to pleasant experiences in Session 2). Breathing space, 3 min 3 times a day at scheduled times. Appreciation exercise before bed. Suggestions for daily life.
4	Importance of the sleep-wake rhythm. Education on how to improve sleep hygiene, with practical proposals.	Keep a daily sleep record that indicates the implementation of recommendations and the results.	Change from trying to avoid, escape, or eliminate unpleasant experiences to approaching them with curiosity and interest.	Sedentary meditation on unpleasant experiences, 30 min with audio every day. Record unpleasant experiences (analogous to pleasant experiences in session 2). Breathing space, 3 min 3 times a day at scheduled times. Appreciation exercise before bed. Suggestions for daily life.
5	Importance of regular and safe exposure to sunlight, with practical recommendations.	Receive at least 1 h of sunlight per day and keep a daily record that includes adherence to the safety guidelines.	Change from needing things to be different to simply letting them be as they are.	Sedentary meditation (sounds and thoughts) with audio every day. Breathing space, 3 times a day at scheduled times and in difficult moments (coping). Meditative walk (walking meditation) as a complement or alternative to seated meditation. Suggestions for informal practice.
6	Effect of negative ruminations in maintaining depression. Learning strategies to detect and stop this behavior.	Keep a daily record of the estimated time of depressive ruminations, strategies used to stop this behavior, and outcomes.	Change from considering thoughts as true and real to considering them as mental processes that may not correspond to reality.	Sedentary meditation (sounds, thoughts, emotions, and body), also without audio support. Breathing space, 3 times a day at scheduled times and in difficult moments (coping version). Attention to and recording of repetitive thoughts. Suggestions for daily life.
7	Importance of contact with nature, and practical recommendations for improvements.	Schedule and engage in at least 1 outing to a natural environment (forest, mountain, beach, or park), and increase these outings, if possible.	Change from treating yourself harshly to caring for yourself with affection and compassion.	Choose between different practices (body scan, conscious stretching, walking meditation, seated meditation, meditation with difficulties, focusing on breathing, and mountain meditation) to create your own program for the week. Devise an action plan for difficult moments (cognitive vulnerability). Breathing space, 3 times a day at scheduled times and in difficult moments (coping version).
8	Review of all strategies and a final opportunity to recommend their combined long-term use.	Proposals for maintaining all these strategies in the future.	Plan a future by living mindfully.	Proposals for maintaining all these strategies in the future.

The eight 3-hour group sessions administered to the active control group (Table 1) will review an assignment of homework on the following topics about depression and mindfulness: symptoms, causes, course, and treatment of depression; concept of full attention; changing from living on “automatic pilot” to living consciously and deliberately; observations of breathing and the body; changing the form of relating to experiences through thought to feeling them directly; labeling different experiences (pleasant, unpleasant); changing from delving into the past and anticipating the future to being fully present in the moment; changing from trying to avoid, escape, or eliminate unpleasant experiences to approaching them with curiosity and interest; changing from needing things to be different to simply letting them be as they are; changing from considering thoughts as true and real to considering them as mental processes that may not correspond to reality; changing from treating yourself harshly to caring for yourself with affection and compassion; planning a future by living mindfully.

### 2.6 Outcomes measures

The primary outcome measure will be the severity of depression based on the Beck depression inventory-II (BDI-II). The secondary outcome measures will be the scores from scales that rate global clinical impression, quality of life, comorbidities, social support, mindfulness skills, physical activity, adherence to the Mediterranean diet, severity of insomnia, self-compassion, and acceptance and action.

#### 2.6.1 Beck depression inventory-II (BDI-II)

The primary outcome is depression severity, and will be measured using the BDI-II.^[38]^ This is a self-report that measures the severity of depression using 21 multiple-choice questions, with each answer scored from 0 to 3. The Spanish version of the BDI-II was validated and has a high reliability (Cronbach alpha = 0.89).^[39]^ The standardized cutoffs are: 0 to 13 (minimal depression); 14 to 19 (mild depression); 20 to 28 (moderate depression); and 29 to 63 (severe depression).

#### 2.6.2 Global clinical impression (GCI)

The CGI was developed for use in clinical trials sponsored by the *National Institute of Mental Health* as a brief, stand-alone assessment of the clinician's view of a patient's global functioning before and after initiating a study medication.^[40]^ The CGI-Severity (CGI-S) asks the clinician 1 question: “Considering your total clinical experience with this particular population, how mentally ill is the patient at this time?” The answer is rated on a 7-point scale (1, normal, not at all ill; 2, borderline mentally ill; 3, mildly ill; 4, moderately ill; 5, =markedly ill; 6, severely ill; 7, among the most extremely ill patients). This rating is based on observed and reported symptoms, behaviors, and function during the past 7 days. The CGI-S score is useful because it can track clinical progress over time and the score correlates with the scores from longer and more time-consuming rating instruments used to assess patients with a diverse psychiatric diagnoses.^[41]^

#### 2.6.3 Three-level European quality of life-5 dimensions questionnaire (EQ-5D-3L)

The health-related quality of life will be measured using the EQ-5D-3L,^[42,43]^ and this score will then be used to calculate the quality-adjusted life-years during the monitoring period by adjusting the length of time affected by the health result by the utility value. This scale measures 5 health dimensions (mobility, self-care, usual activities, pain/discomfort, and anxiety/depression) each of which is rated at 3 levels (no problems, slight problems, or moderate/severe problems). The EQ-5D-3L also records a patient's self-rated health using a 20-cm vertical visual analogue scale (VAS), in which the endpoints are labeled “The best health you can imagine” and “The worst health you can imagine.” This VAS can be used as a quantitative health outcome measure that reflects the patient's own judgment. A patient marks the point on the vertical line that best reflects his or her assessment of current global health status.^[44]^ Cronbach's alpha coefficient for this scale has been calculated in some disease-specific populations. We highlight the study by Seoane et al,^[45]^ in which the overall value was 0.788. One general population study provided an overall mean estimate of the Minimum Important Difference for the EQ-5D was 0.074.^[46]^

#### 2.6.4 International classification of diseases (ICD-10)

The presence of comorbidities and chronic diseases will be recorded using the ICD-10.^[47]^ We estimate that approximately 50% of patients will present with at least 1 comorbidity.^[48]^ Anthropometric measures (weight, size, and waist circumference) will also be recorded.

#### 2.6.5 Medical outcomes study social support survey (MOS-SS)

Social support will be measured using the MOS-SS,^[49]^ a self-report instrument that has 4 subscales (emotional/informational, tangible, affectionate, and positive social interaction) and an overall functional social support index. This scale has good reliability (Cronbach's alpha ≥ 0.91) and is stable over time. It consists of 19 items, each of which is rated using a 5-point Likert scale. Higher scores indicate greater support. The Spanish language version of the MOS-SS has been validated.^[50]^

#### 2.6.6 Five facet mindfulness questionnaire (FFMQ)

The FFMQ^[51]^ is a self-report questionnaire that assesses 5 facets of mindfulness: observation, description, aware actions, nonjudgmental inner experience, and nonreactivity. It contains 39 items, each of which is rated from 1 (“never or very rarely true”) to 5 (“very often or always true”). The total score is the sum of the direct and reverse-scored items. The Spanish language version has been validated and has a Cronbach alpha of 0.88.^[52]^

#### 2.6.7 Physical activity based on the international physical activity questionnaire-short form (IPAQ-SF)

Physical activity will be measured using IPAQ-SF.^[53]^ This scale assesses the levels of habitual physical activity over the previous 7 days. It has 7 items and records activity on 4 intensity levels (vigorous intensity, moderate intensity, walking, and sitting). A validated Spanish version is available.^[54]^ This scale has good reliability and acceptable validity for the measurement of total and vigorous physical activity, but poor validity for moderate activity.^[55]^

#### 2.6.8 Mediterranean diet adherence screener (MEDAS)

Adherence to the Mediterranean diet will be measured using the 14-item MEDAS, developed by the PREDIMED study group.^[56]^ This scale records food consumption habits. It considers the consumption of olive oil as the main source of cooking fat, white meat and red meat, servings of vegetables, portions of fruit, red meat or sausages, animal fat, sugar-sweetened beverages, red wine, pulses, fish, commercial pastries, and dressing foods with a traditional sauces consisting of tomatoes, garlic, onions, or leeks sautéed in olive oil. The total score ranges from 0 to 14, with a higher score indicating better adherence.^[57]^

#### 2.6.9 Insomnia severity index (ISI)

The ISI^[58]^ will be used to assess insomnia. This is a self-report instrument that measures a patient's perception of nocturnal and diurnal symptoms of insomnia: difficulties initiating sleep, staying asleep, early morning awakening, satisfaction with current sleep pattern, interference with daily functioning, noticeability of impairment attributed to a sleep problem, and degree of distress or concern caused by a sleep problem. This scale has 7 items, with each answer ranging from 0 to 4, and an overall score ranging from 0 to 28. The Spanish version of the ISI^[59]^ has adequate internal consistency (Cronbach alpha = 0.82).

#### 2.6.10 Self-compassion scale (SCS)

The original SCS^[59]^ has 26 items that measure 6 components of self-compassion (with reverse coding of negative aspects): self-kindness, self-judgment, common humanity, isolation, mindfulness, and over-identification.^[60]^ Each item is rated on a 5-point scale that ranges from 1 (“almost never”) to 5 (“almost always”). The Spanish version of the short form of the SCS (12 items) is a valid and reliable instrument for evaluation of self-compassion in the general population.^[61]^

#### 2.6.11 Acceptance and action questionnaire-II (AAQ-II)

The AAQ-II^[62]^ is a general measure of experiential avoidance and psychological inflexibility which consists of 7 statements, each of which is rated from 1 (“never true”) to 7 (“always true”). This scale measures the unwillingness to experience unwanted emotions and thoughts and the inability to be in the present moment and commit to values-directed actions when experiencing psychological events that could undermine them. The Spanish version of the AAQ-II has good internal consistency, in that Cronbach's alpha was 0.88; construct validity, in that the factor analysis led to a 1-factor solution; discriminant validity, in that the score discriminated between clinical and nonclinical samples; and external validity, in that there were strong correlations with a wide range of psychological symptoms, quality of life, and other psychological constructs.^[63]^

### 2.7 Participant timeline

We will collect patient data using questionnaires administered at baseline (week-0), immediately after the intervention (week-8), at month-6, and at month-12 (Table 2).

**Table 2:** Timeline for enrolment, allocation, interventions, and assessments.

	Study period				
	
	Enrolment	Intervention	Post-allocation		Close-out
				
Timepoint	−1 wk	0	8 wk	6 mo	12 mo
Enrolment					
Eligibility screen	X				
Informed consent	X				
Allocation		x			
Interventions					
MBCT intervention		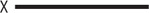	x		
Lifestyles intervention		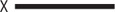	x		
Placebo		x			
Assessments					
Chronic conditions (ICD-10), BDI-II	x				
BDI-II, GCI, EQ-5D-3L			x	x	X
MOS-SS, IPAQ-SF, MEDAS, ISI			x	x	x

### 2.8 Sample size

Scientific evidence suggests that a 17% reduction in the BDI-II score^[38]^ is clinically relevant.^[64]^ A previous study by our team that examined psychiatric outpatients indicated the average BDI-III score at the beginning of the study was 24.5 (SD = 9.8).^[9]^ Thus, we considered a reduction of 6 points or more to indicate a clinically significant benefit. We estimate a maximum dropout rate of 25%. Accepting an α risk of 0.05 and a β risk of 0.15 in a bilateral contrast, 60 subjects will be required for each group. Thus, the total sample size will be 180.

### 2.9 Recruitment

Patients from the Balearic Islands who experienced a TRD episode will be recruited through cooperation with mental health workers and applications will be submitted via social media. To ensure the patients meet the inclusion criteria, the assessors will administer the MINI questionnaire by telephone, as with all the other tests.^[37]^ If a participant is eligible, the researcher will administer the baseline questionnaire. Recruitment and baseline assessments will be performed until the sample size is 180. All collected information will be processed as stipulated in current legislation regarding the protection of personal data.

### 2.10 Assignment of interventions

Eligible patients will be randomized once they agree to participate and provide written informed consent. After the enrolment visit, each patient will be randomly assigned to the MBCT Intervention, Lifestyles Intervention or Placebo group in a 1:1:1 ratio. Randomization will be performed using a computer-generated random assignment sequence with a block randomization method and Random Allocation software (v.2.0). A hospital unit unrelated to this clinical trial will perform the randomization of patients to the different study groups.

### 2.11 Statistical analyses

All researchers conducting the data analysis will be blinded to group allocations. All eligible randomized patients who were evaluated at least once using the efficacy indicators will be defined as the “full analysis set”; all eligible randomized patients who attend at least 80% of the scheduled sessions will be defined as the “per-protocol set.” The effectiveness measurements will be evaluated mainly by intention-to-treat analysis using the full analysis set and supplemented by per-protocol analysis.

Numerical variables with normal distributions will be presented as means ± standard deviations, and categorical variables as absolute and relative frequencies. Numerical variables with skewed distributions will be presented as medians, minima, and maxima. Analysis of variance will be used to compare the 3 groups at multiple time points. In the main analysis, missing data will be replaced using multiple imputation. Complete-case analysis will be also conducted as a sensitivity analysis to assess the robustness of results from the multiple imputation procedure. Hypothesis testing will be performed using a 2-sided test and presented with 95% confidence intervals. A *P* value less than .05 will indicate statistical significance. All analyses will be performed using SPSS version 21.0 and STATA version 16.1.

## 3 Discussion

Mental health professionals have the responsibility to adapt their health care interventions to health emergency situations, such as the current COVID-19 pandemic, and must continue to offer demonstrably useful therapeutic alternatives to their patients. Patients with TRD are among the neediest in these circumstances, because provision of face-to-face care is very difficult. This study will examine the effect of an intervention that attempts to help patients with TRD to modify their lifestyles by using information and communications technologies (ICTs) and videoconferencing instead of traditional face-to-face interactions. To assess the efficacy of lifestyle modification, we will compare this intervention group with 2 control groups: a placebo control group, which will receive minimal assistance (written recommendations for a healthy lifestyle), and an active control group, which will participate in an iMBCT program.

The time commitment is the same for the active control (iMBCT) group and the intervention (lifestyle program) group, and iMBCT has been used successfully for patients with TRD in face-to-face settings. We hypothesize that at the end of the 12-month follow-up, the lifestyle program group will show greater adherence to the intervention and will therefore experience greater improvement than the other 2 groups. If this Internet-based multimodal lifestyle intervention program is useful as an adjunct to the pharmacological treatment of depression, this approach should be considered for the management of patients with TRD in similar emergency situations.

However, there are certain limitations that we must accept. All assessment instruments are self-applied to avoid possible assessor bias. However, it is not possible to blind study subjects in the 3 groups. Therefore, if patients aim to please the research team this could lead to response bias. To reduce this bias, we will inform all patients that each complementary treatment may be effective and will not explain our hypothesis regarding the potential greater long-term efficacy of the lifestyle intervention program. A second possible limitation is that certain elderly participants may have difficulties using ICTs. In these cases, we will recommend the assistance by family members. Another possible limitation is our uncertainty of the extent to which the COVID-19 pandemic will affect our study. In particular, a high infection rate could make it difficult to recruit participants and could also increase their stress, due to its adverse effects on health, income, and social factors, and thus hinder the efficacy of the treatment.

Despite these possible limitations, we believe it is urgent and necessary to develop an effective new intervention for patients with TRD who are facing uncertain and difficult situations, such as the COVID-19 pandemic.

## Acknowledgments

The authors are grateful to the field staff and participants of this study.

## Author contributions

**Conceptualization:** Capilla Navarro, Aina M Yáñez, Aurora Garcia, Rocio Gomez-Juanes, Mauro Garcia-Toro.

**Investigation:** Francisco Gazquez, Jose Antonio Marino, Andrea Seguí, Aurora Garcia.

**Methodology:** Capilla Navarro, Aina M Yáñez, Aurora Garcia, Rocio Gomez-Juanes, Mauro Garcia-Toro.

**Supervision:** Capilla Navarro, Mauro Garcia-Toro.

**Writing – original draft:** Capilla Navarro, Aina M Yáñez, Aurora Garcia, Rocio Gomez-Juanes, Mauro Garcia-Toro.

**Writing – review & editing:** Andrea Seguí, Francisco Gazquez, Jose Antonio Marino, Olga Ibarra, Maria J. Serrano-Ripoll, Miquel Bennasar-Veny, Joan Salva, Bárbara Oliván, Miquel Roca, Margalida Gili.
